# Editorial: Vitamin D and women’s health across the lifespan

**DOI:** 10.3389/fendo.2026.1949614

**Published:** 2026-09-23

**Authors:** Grigoris Effraimidis, Konstantinos Michalakis, Hana Fakhoury, Fatme Al Anouti, Spyridon Karras

**Affiliations:** 1 Faculty of Medicine, School of Health Sciences, University of Thessaly, Larissa, Greece; 2 Department of Endocrinology and Metabolic Diseases, Larissa University Hospital, Larissa, Greece; 3 Department of Obesity and Metabolism, Endocrine Practice, Athens, Greece; 4 College of Medicine, Alfaisal University, Riyadh, Saudi Arabia; 5 Department of Health Sciences, College of Natural and Health Sciences, Zayed University, Abu Dhabi, United Arab Emirates; 6 1st Laboratory of Pharmacology, School of Medicine, Aristotle University of Thessaloniki, Thessaloniki, Greece

**Keywords:** lifespan, pregna-4,9(11)-diene-3-one-20-carboxylic acid methyl ester, reproduction, vitamin D, women

Vitamin D holds an unusual position in contemporary medicine. Its importance for calcium-phosphate homeostasis and skeletal health is undisputed, whereas its proposed reproductive, metabolic, immune, and developmental effects remain the subject of intense investigation. Recent guidance reflects this tension: empiric vitamin D supplementation is recommended during pregnancy, but routine 25-hydroxyvitamin D [25(OH)D] testing is discouraged in otherwise healthy populations because outcome-specific thresholds and benefits of screening remain uncertain (1). Even the definition of deficiency is contentious: the 20 ng/mL versus 30 ng/mL (50 versus 75 nmol/L) cutoff debate remains unresolved, and persistent discrepancies between immunoassays and LC-MS/MS limit cross-study comparability. Similarly, large randomized trials have tempered expectations of broad extraskeletal benefits in vitamin D-replete individuals, thus reinforcing the need to prevent and correct genuine deficiency (2). Against this backdrop, this Research Topic examined a more clinically useful question: not simply whether vitamin D “works,” but rather, in whom, at which life stage, and for which outcome or purpose.

Reproductive medicine is the dominant theme of this Research Topic and illustrates why population and endpoint matter. Luo et al. found no association between serum 25(OH)D and embryo quality, implantation, or clinical pregnancy in women with normal ovarian reserve undergoing a first IVF/ICSI cycle. In contrast, Wang et al. reported lower clinical pregnancy rates among vitamin D-deficient women aged ≥35 years, along with reduced expression of the endometrial receptivity marker HOXA10, suggesting that age and endometrial biology may modify the association.

Between the collection of these articles and the present review, polycystic ovary syndrome (PCOS) has been officially renamed to Polyendocrine Metabolic Ovarian Syndrome (PMOS) by a global panel of experts because the new name better reflects the condition’s nature as a whole-body disorder affecting hormones, metabolism, and ovulation, rather than just an issue with ovarian cysts. Thus, among women with PMOS, Yu et al. observed a lower clinical pregnancy rate with vitamin D deficiency, although live birth and embryological outcomes were not significantly different. These findings align with the mechanistic framework developed by Subakathulla et al., who situated vitamin D within an insulin resistance–inflammation–oxidative stress axis relevant to PMOS. Mechanistic plausibility, however, is not the same as clinical synergy: combining vitamin D with myo-inositol or melatonin remains a hypothesis that only factorial, phenotype-stratified trials can test.

Ovarian reserve is another example of the distinction between association and therapeutic efficacy. Zhang et al. found that women with diminished ovarian reserve had lower serum and follicular-fluid 25(OH)D concentrations and that higher levels were associated with improved oocyte retrieval and normal fertilization rates. However, a meta-analysis by Fang et al., incorporating 11 randomized trials and 992 women, found no significant overall effect of vitamin D supplementation on anti-Müllerian hormone concentrations. Taken together, these studies suggest that vitamin D status may identify a subgroup or biological milieu associated with reproductive performance, but supplementation should not be presented as a means of restoring the follicular pool. Anti-Müllerian hormone is an intermediate biomarker, and future trials should prioritize cumulative pregnancy and live birth while accounting for age, PMOS phenotype, ovarian reserve, baseline deficiency, season, adiposity, and assay methodology. This measured interpretation is consistent with previous reviews of vitamin D in fertility and PMOS (3).

Pregnancy extends the question from maternal health to intergenerational outcomes. In 2,742 pregnancies assessed using liquid chromatography-tandem mass spectrometry, Zheng et al. reported a high prevalence of deficiency, particularly in early gestation, an association between early deficiency and gestational hypothyroidism, and a greater short-term increase in 25(OH)D with vitamin D3 than with vitamin D2. The unexpected inverse association between late-pregnancy deficiency and preterm birth appropriately cautions against the causal interpretation of observational findings. The KLOTHO birth cohort reported by Karras et al. moves beyond total 25(OH)D by profiling eight maternal-neonatal vitamin D metabolites and relating cord-blood patterns to anthropometry at 10–11 years of age. Although several directional signals emerged, none remained robust after correction for multiple testing, except for a strong tendency to an inverse association of Vitamin D with later BMI and triceps skinfold thickness of offspring. This result highlights the value of long-term follow-up, metabolite-specific measurement, and transparent control of multiplicity and underscores how much remains unknown about the developmental consequences of maternal vitamin D status (4).

The Research Topic further broadens the field beyond reproduction. In women practicing Orthodox religious fasting, Karras et al. showed that 2,500 IU/day of vitamin D3 corrected low vitamin D status but did not significantly change leptin or interleukin-6 concentrations. In a prospective mixed-sex cohort with obesity and type 2 diabetes, Hoffmann et al. observed modest improvements in fasting glucose and HbA1c with 4,000 IU/day, without changes in body weight or blood pressure; the non-randomized design precludes causal inference. El Shamieh et al. identified a strong cross-sectional association between vitamin D deficiency and *Helicobacter pylori* infection, accompanied by differences in sphingolipid and other lipid pathways in an exploratory metabolomics subset. These findings provide hypotheses linking vitamin D, infection, and metabolism; however, reverse causation and residual confounding remain plausible.

Finally, Qahtan et al. reviewed the genetic and epigenetic determinants of vitamin D metabolism, including variations in *GC*, *CYP2R1*, *CYP27B1*, *CYP24A1*, and *VDR*. Their synthesis offers a potential explanation for the heterogeneous results across this Research Topic: identical doses and similar total 25(OH)D concentrations may not produce identical tissue exposure or biological responses. Precision nutrition is therefore an attractive direction, but clinical implementation currently exceeds the evidence. Genetic variants explain only part of interindividual variability, and the majority of epigenetic findings remain inconclusive.

Collectively, these 12 articles argue against both therapeutic nihilism and indiscriminate supplementation. Vitamin D deficiency should be corrected when present, and vitamin D should be administered for established clinical indications. However, it should not be promoted as a universal remedy for infertility, PMOS, metabolic dysfunction, inflammation, or adverse pregnancy outcomes. The next phase of research requires standardized LC-MS/MS-based phenotyping, harmonized definitions, adequate representation across ethnicities and life stages, and trials enriched for participants most likely to benefit. Clinically meaningful endpoints, especially live birth, maternal complications, offspring health, and patient-centered outcomes, must take precedence over isolated surrogate markers. The relative scarcity of studies in perimenopausal and older women also identifies an important gap in a collection explicitly spanning the lifespan.

The central message is therefore one of context: vitamin D is neither merely a skeletal nutrient nor a universal endocrine therapy. Its effects are likely to depend on baseline status, reproductive and metabolic phenotype, timing, tissue biology, and genetic-environmental interactions. Defining these dependencies is the pathway from association to precision care.
